# Resectability, Resections, Survival Outcomes, and Quality of Life in Older Adult Patients with Metastatic Colorectal Cancer (the RAXO-Study)

**DOI:** 10.3390/jcm12103541

**Published:** 2023-05-18

**Authors:** Kaisa Lehtomäki, Leena-Maija Soveri, Emerik Osterlund, Annamarja Lamminmäki, Aki Uutela, Eetu Heervä, Päivi Halonen, Hanna Stedt, Sonja Aho, Timo Muhonen, Annika Ålgars, Tapio Salminen, Raija Kallio, Arno Nordin, Laura Aroviita, Paul Nyandoto, Juha Kononen, Bengt Glimelius, Raija Ristamäki, Helena Isoniemi, Pia Osterlund

**Affiliations:** 1 Faculty of Medicine and Health Technology, Tampere University, Arvo Ylpön Katu 34, 33520 Tampere, Finland; 2 Department of Oncology, Tays Cancer Centre, Tampere University Hospital, Teiskontie 35, 33520 Tampere, Finland; 3 Department of Oncology, Comprehensive Cancer Center, Helsinki University Hospital, 00290 Helsinki, Finland; 4 Department of Oncology, Clinicum, University of Helsinki, 00014 Helsinki, Finland; 5 Home Care, Joint Municipal Authority for Health Care and Social Services in Keski-Uusimaa, 05850 Hyvinkää, Finland; 6 Transplantation and Liver Surgery, Abdominal Center, Helsinki University Hospital, 00290 Helsinki, Finland; 7 Department of Immunology, Genetics and Pathology, Uppsala University, 75185 Uppsala, Sweden; 8 Department of Oncology, Kuopio University Hospital, Puijonlaaksontie 2, 70210 Kuopio, Finland; 9 Faculty of Health Sciences, University of Eastern Finland, Yliopistonranta 1A, 70210 Kuopio, Finland; 10 Department of Surgery, Clinicum, University of Helsinki, 00014 Helsinki, Finland; 11 Department of Oncology, Turku University Hospital, Hämeentie 11, 20520 Turku, Finland; 12 Department of Oncology, University of Turku, Kiinanmyllynkatu 10, 20520 Turku, Finland; 13 Department of Oncology, South Carelia Central Hospital, Valto Käkelän Katu 1, 53130 Lappeenranta, Finland; 14 Department of Oncology, Oulu University Hospital, Kajaanintie 50, 90220 Oulu, Finland; 15 Department of Oncology, Kanta-Häme Central Hospital, Ahvenistontie 20, 13530 Hämeenlinna, Finland; 16 Department of Oncology, Päijät-Häme Central Hospital, Keskussairaalankatu 7, 15850 Lahti, Finland; 17 Docrates Cancer Centre, Docrates Hospital, Saukonpaadenranta 2, 00180 Helsinki, Finland; 18 Department of Oncology, Central Finland Central Hospital, Keskussairaalantie 19, 40620 Jyväskylä, Finland; 19 Department of Gastrointestinal Oncology, Tema Cancer, Karolinska Universitetssjukhuset, Eugeniavägen 3, 17176 Solna, Sweden; 20 Department of Oncology-Pathology, Karolinska Institutet, Solnavägen 1, 17177 Solna, Sweden

**Keywords:** older adults, metastatic colorectal cancer, metastasectomy, liver resection, lung resection, cytoreductive surgery, local ablative therapy, oncological treatment, health-related quality of life

## Abstract

Older adults are underrepresented in metastatic colorectal cancer (mCRC) studies and thus may not receive optimal treatment, especially not metastasectomies. The prospective Finnish real-life RAXO-study included 1086 any organ mCRC patients. We assessed repeated centralized resectability, overall survival (OS), and quality of life (QoL) using 15D and EORTC QLQ-C30/CR29. Older adults (>75 years; n = 181, 17%) had worse ECOG performance status than adults (<75 years, n = 905, 83%), and their metastases were less likely upfront resectable. The local hospitals underestimated resectability in 48% of older adults and in 34% of adults compared with the centralized multidisciplinary team (MDT) evaluation (*p* < 0.001). The older adults compared with adults were less likely to undergo curative-intent R0/1-resection (19% vs. 32%), but when resection was achieved, OS was not significantly different (HR 1.54 [CI 95% 0.9–2.6]; 5-year OS-rate 58% vs. 67%). ‘Systemic therapy only’ patients had no age-related survival differences. QoL was similar in older adults and adults during curative treatment phase (15D 0.882–0.959/0.872–0.907 [scale 0–1]; GHS 62–94/68–79 [scale 0–100], respectively). Complete curative-intent resection of mCRC leads to excellent survival and QoL even in older adults. Older adults with mCRC should be actively evaluated by a specialized MDT and offered surgical or local ablative treatment whenever possible.

## 1. Introduction

Colorectal cancer (CRC) is the third most common cancer in the western world and the second most common cause of cancer death, and more than half of the patients are over 70 years old [1]. Radical resection of the primary tumor, and either resections and/or local ablative therapies (LAT) of metastases are the only potentially curative treatment options in metastatic CRC (mCRC) [2,3]. Due to an increasing number of comorbidities and diminishing physiological reserves in older age, there are concerns that older adults may not tolerate aggressive treatment regimens the same as younger patients, and that their health-related quality of life (HRQoL) is deteriorated if treated too aggressively [4]. Liver resection with curative intent for CRC metastases is feasible also in older adults, though with higher perioperative risks than in younger patients [5,6,7]. A systematic review and meta-analysis reported increased postoperative morbidity and mortality rates when liver resections were performed in patients over 75 years old compared with younger patients, but there were no differences in overall survival (OS) or disease-free survival (DFS) [8]. However, less is known about extrahepatic metastasectomies in older adults.

Older adults with mCRC are underrepresented in clinical trials, and there are also studies showing that they are less frequently discussed at multidisciplinary teams (MDTs), may not always be offered the same treatment options as younger patients, and may more often be treated with non-curative treatment strategies [9,10,11,12]. Older adults value an active role and shared decision-making with an opportunity to express their preferences on treatment options [13], and often weigh good HRQoL, maintenance of functional status, and independency over maximal prolongation of life [14,15]. Thus, in addition to classical endpoints in oncological trials such as survival, HRQoL measuring functional, social and emotional issues is especially valuable in studies exploring treatment decisions of older adults, ensuring that treatment decisions are performed according to patients’ needs and wishes [11].

We recently reported from a nationwide prospective Finnish RAXO-study that repeated centralized MDT assessment in real-world mCRC patients generates high resectability and resection rates with impressive survival [16]. The aim of the current study was to evaluate whether there were differences in resectability of CRC metastases when assessed with centralized or local MDT, resection and/or LAT, or outcome between older adults (over 75 years) and adults, and how the older patients perceived their HRQoL during the intensive treatments often needed prior to and after the resection and/or LAT, and during palliative systemic therapy.

## 2. Materials and Methods

This study was a prespecified old age substudy of the prospective nationwide investigator-initiated RAXO-study (NCT01531621, EudraCT2011-003158-24), which was conducted at all Finnish hospitals treating mCRC. During the inclusion period, 2012–2018, there was no CRC screening conducted in Finland. The primary objective of the prospective study was to evaluate the impact of repeated centralized MDT assessment on technical resectability of metastases, upfront and after conversion therapy, performed resections and/or LAT, and outcomes after resection. The RAXO-study is described in detail elsewhere [16]. Inclusion criteria were histologically confirmed synchronous or metachronous mCRC; patient scheduled for first-line systemic therapy, that is, wanted to have oncologic treatment for mCRC; age over 18 years, and signed written informed consent obtained according to Good Clinical Practice. The ethics committee at Helsinki University Hospital (242/13/03/02/2011) and each hospital approved the study.

Baseline resectability was assessed locally by organ-specific MDTs, organ-specific surgeons (hepatobiliary, thoracic etc.), bowel MDT (without organ-specific surgeons or interventional radiologists), or oncologists. To assess resectability centralized, all patients were evaluated at Helsinki University Hospital by a specialized MDT, which consisted of experienced liver surgeons and abdominal radiologists and other specialists; for example, medical oncologists, radiation oncologists, gastrointestinal surgeons, thoracic surgeons, cytoreductive surgeons, gynecologists, thoracic radiologists, pathologists, and PET/CT specialists, as needed. The centralized MDT assessed technical resectability of liver, lung, and other metastases based upon available radiology, mostly high-quality body computed tomography, and, as needed, magnetic resonance imaging and positron emission tomography, at baseline and repeated up to twice during the first-line systemic therapy; detailed description of the process in [16]. This second opinion on technical resectability and conversion was reported online via www.raxo.fi to the local hospital. Treatment decisions regarding resection and/or LAT (such as thermoablation with radio-frequency or microwave ablation or stereotactic body radiotherapy—SBRT) were always made locally with full knowledge of patient operability, comorbidities, etc. Ultimately, patients were reassessed by local organ-specific MDTs and resections were either performed locally or the patient was referred to a tertiary unit. The postoperative follow-up was performed according to the study protocol.

Patients were upfront categorized into three groups both by local and centralized MDT: resectable, borderline, or unresectable. The upfront borderline and unresectable patients were reassessed twice and ultimately classified as converted, unconverted, or never resectable. The reasons for non-resectability and why resectable patients were not resected were recorded.

Each hospital used its own standard treatment protocols based on the National Comprehensive Cancer Network and European Society for Medical Oncology guidelines from 2012 onwards. Systemic therapy was given until disease progression or toxicity occurred, or resectability had been achieved.

### 2.1. Quality of Life Questionnaires

For HRQoL evaluation, four different HRQoL questionnaires were used: the generic 15D [17] and EQ-5D-3L [18], which produce both index and profile data, and the disease-specific EORTC QLQ-C30 [19] and QLQ-CR29 [20], of which QLQ-C30 produces both index (global health status—GHS) and profile measures, and QLQ-CR29 colorectal cancer-specific profile measures. The HRQoL assessments are previously described in detail in [21]. 

**Figure 1:**
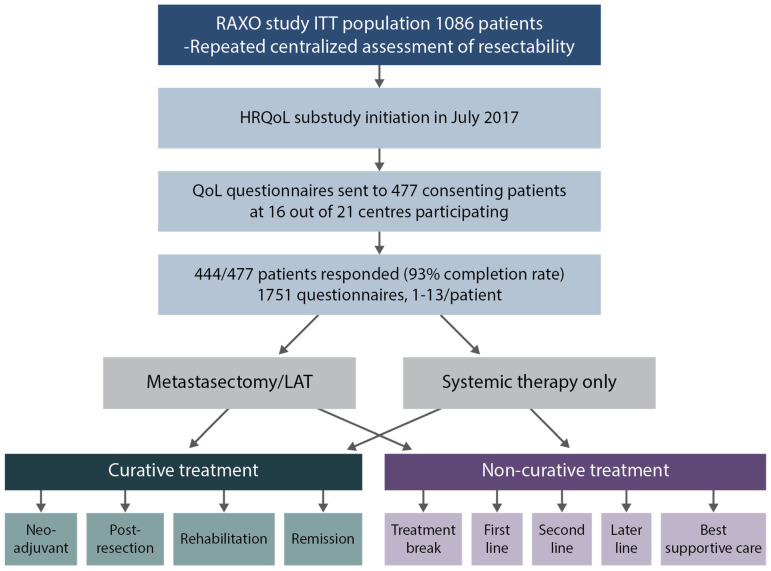
Study design, patient flow, Health-Related Quality of Life (HRQoL) questionnaires, and intervention with resectability assessments and metastasectomy, and/or local ablative therapy (LAT) rates at a centralized multidisciplinary team (MDT) conference at a tertiary hospital in the Finnish nationwide RAXO-study.

The HRQoL data were collected multi-cross-sectionally (maximum 13 times) and analyzed according to treatment phases (Figure 1). Questionnaires were given to patients at the hospital or sent out by mail. Patients were instructed to fill in the questionnaires just before a response evaluation and/or a doctor’s appointment. Time points were, thus, not treatment-phase-dependent or scheduled to baseline, at certain timepoints during a treatment phase, or after progression. Treatment phases were defined as curative if the patient was/became resectable and metastasectomy/LAT was performed, and divided into neoadjuvant/conversion, postoperative (during the first 6 months after resection/LAT, including adjuvant therapy), rehabilitation (without treatment 6–18 months from an R0/1-resection/LAT or complete response to systemic therapy only), and remission (disease-free for more than 18 months from the last metastasectomy/LAT or CR). Patients completed the questionnaires in remission phase at a median of 38 months (maximum 94 months) from the last metastasectomy/LAT. The non-curative treatment phases were when systemic therapy was given with the goal of life-prolongation and palliation, and no curative metastasectomy/LAT could be performed (despite conversion aim in some patients), and included first-line, second-line, and later-line systemic treatment. For details see [21].

### 2.2. Statistical Analysis

SPSS version 27.0, Armonk, NY, USA was used for the statistical analyses. Results are presented as mean values with standard deviations and 95% confidence intervals (CI) for HRQoL scores in each treatment phase. For patient characteristics, proportions and median with range or interquartile range (IQR) are presented. Comparisons were performed with the non-parametric Mann–Whitney test for two-group-independent samples and the Wilcoxon signed-rank test for paired samples. Minimal clinically important difference (MID) was used with cutoffs as described in Ref. [21]. We calculated proportions for the key demographic characteristics with Chi-square tests with Bonferroni correction per variable. Survival was assessed in the intention-to-treat populations by Kaplan–Meier estimates and Cox proportional hazard regression with hazard ratios (HR) and corresponding 95% confidence intervals (CI). In the multivariable model age, Eastern Cooperative Oncology Group performance status (ECOG PS), treatment group, Charlson comorbidity index (CCI), primary tumor location, primary tumor operation, and mutational status were included in accordance with the analysis plan. The cutoff date for survival status was 30 April 2020. OS was calculated from the diagnosis of mCRC to death from any cause or censored at last follow-up. Two-sided *p* < 0.05 was considered statistically significant. No adjustments for potential multiplicity were performed.

## 3. Results

### 3.1. Patient Characteristics

From 2012 to 2018, the national RAXO-study recruited 1086 treatable mCRC patients, of which, 181 (17%) were older adults (>75 years) and 905 (83%) were adults (age ≤75 years). Patient characteristics are presented in Table 1. Median age was 78 years (range 75–90) in older adults and 64 among adults. There was no gender imbalance between the age groups (59% vs. 61% males). Older adults more often had an ECOG PS 2–3, comorbidities, and a history of other malignancies than adults. Tumor characteristics in older adults showed significantly more metachronous metastases, more often underwent surgery of the primary tumor upfront, and there were more lung and extrapulmonary metastases. Less thrombocytosis was seen among the older adults. There were no statistically significant differences in mutational status, but older adults were less frequently tested for *RAS*/*BRAF* status (Table 1).

### 3.2. Assessment of Resectability

A centralized assessment of technical resectability for all patients regardless of age and local assessment was performed. The MDT was provided with patient details including comorbidities inhibiting operation and whether or not patients wanted to undergo operation, but technical resectability was assessed regardless of those factors.

**Figure 2:**
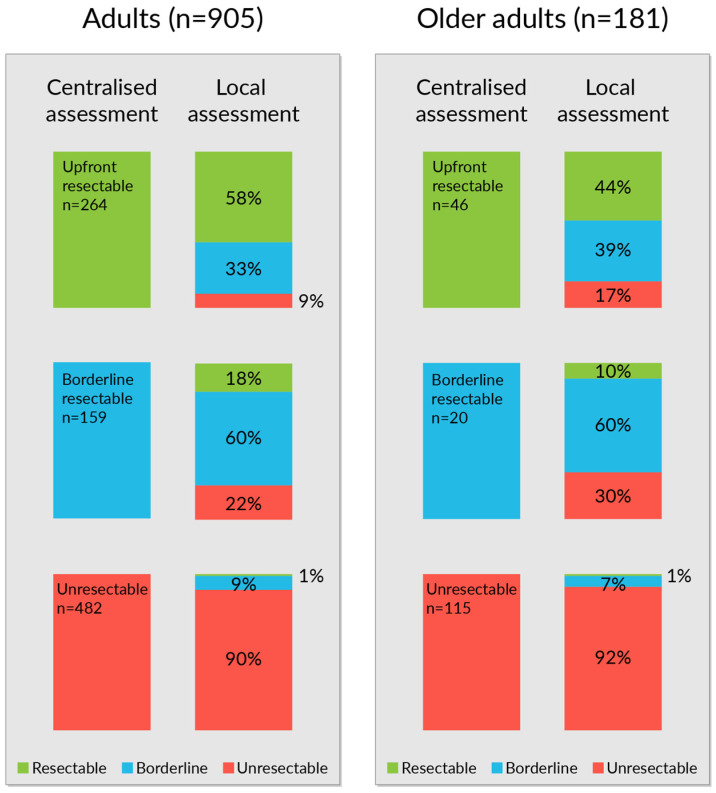
Centralized and local upfront assessment of resectability at diagnosis of metastatic colorectal cancer in adults and older adults.

In the centralized assessment at baseline, older adults had slightly less upfront resectable metastases (25% vs. 29%), less borderline resectable metastases (11% vs. 18%), and, thus, more non-resectable metastases than adults (64% vs. 53%, *p*-value 0.036) (for patient numbers see Figure 2).

The local assessment compared with the centralized assessment underestimated upfront resectability in 42% of adults and in 56% of older adults, and in upfront borderline resectable, an underestimation was seen in 22% and 30%, respectively (*p* < 0.001; Figure 2). An overestimation of resectability in the local assessment compared with the centralized was seen in 8% of unresectable older adults and in 10% of unresectable adults. Concordance for resectable patients was 62–100% for older adults and 72–79% for adults when assessed in local organ-specific MDT or organ-specific surgeon, but only in 0–7% for older adults and in 24–27% for adults when assessed by a local bowel MDT or by a clinical oncologist alone.

Successful conversion with systemic therapy in baseline borderline and unresectable patients at centralized re-assessment was rarer among older adults than adults (10% (13/135) vs. 19% (124/641), *p* = 0.015).

**Figure 3:**
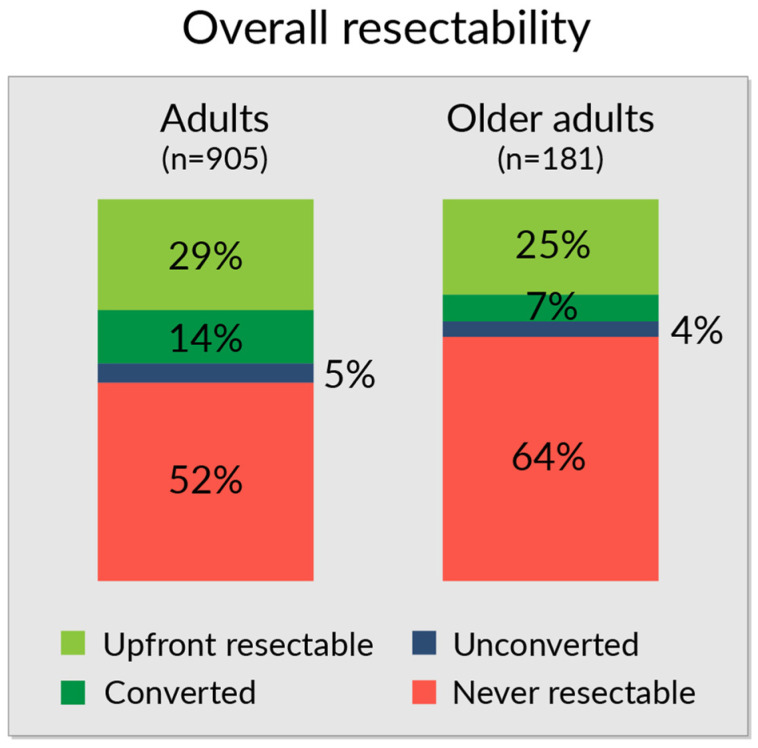
Overall resectability after centralized upfront and two re-assessments during first and second response evaluation with neoadjuvant/conversion therapy in adults (n = 905) and in older adults (n = 181). Upfront resectable in light green, borderline successfully converted in dark green, borderline unconverted in blue, and never resectable (still not resectable) in red.

After three centralized assessments, fewer older adults were considered resectable (either upfront resectable or successfully converted) than adults (32% vs. 43%) whereas never resectable were seen in 64% vs. 52%, respectively (*p* = 0.034, Figure 3).

### 3.3. Resection Rates at Different Metastatic Sites

Both liver and lung procedures, including resections and/or LAT, mostly thermoablation or stereotactic body radiation therapy (SBRT), were significantly less often performed in older adults than in adults (Table 2; 20% vs. 31%; *p* = 0.005 and 4% vs. 8%; 0.044, respectively). No statistical differences in cytoreductive surgery, local, lymphadenectomy, gynecologic, urologic, or other resections were noted. Re-resections with two or more liver, lung, and local procedures were performed as often in older adults as in adults (mean 1.6 resections and/or LAT per patient for older adults and 1.7 for adults). In both groups, progressive disease and comorbidities were the main reasons for not resecting a technically resectable patient, with no differences among age groups.

An R0/1-resection was performed less often in older adults than in adults (19% vs. 32%, *p* < 0.001). Non-radical R2-resections and/or LAT procedures were similar in frequencies (6% in older adults and 7% in adults). Older adults, who were assessed upfront resectable significantly more often had LAT of liver or lung metastases compared with adults (13% vs. 5%; *p* = 0.026).

### 3.4. Systemic Therapy

Systemic therapy in conjunction with metastasectomy and/or LAT was given to 89% (357 of the 399 with resection/LAT), with no difference between older adults and adults (Table 3). Similar proportions also had metastasectomy only without systemic treatment. Adjuvant therapy after metastasectomy/LAT was given in 60% of older adults and in 67% of adults (*p* = 0.374).

The duration of the systemic treatment (including neoadjuvant/conversion, adjuvant, and/or palliative after non-re-resectable relapse) in patients with R0/1-resected tumors was median—8.1 months (IQR 6–13) in older adults and similar—8.4 months (5–17) among adults (*p* = 0.566). In R2/LAT-treated patients, the systemic treatment duration was longer, though without difference between older adults and adults—12.5 months (9–27) vs. 14.2 months (6–24) (*p* = 0.926).

Older adults were more often treated with ‘systemic therapy only’ when looking at all patients (71% vs. 59%, *p* = 0.004) or with best supportive care only; the latter was due to disease progression and loss of ‘treatability’ after inclusion (4% vs. 2% of all; *p* = 0.018). The duration of ‘systemic therapy only’ was shorter for older adults (10.6 months [IQR 4–17] vs. 12.8 months [6–20] for adults; *p* = 0.012). The first treatment intent in ‘systemic therapy only’ patients was disease control in 71% of older adults and in 77% of adults (*p* = 0.130), and unsuccessful neoadjuvant or conversion in 23% and 20%, respectively (Table 3).

### 3.5. Survival

**Figure 4:**
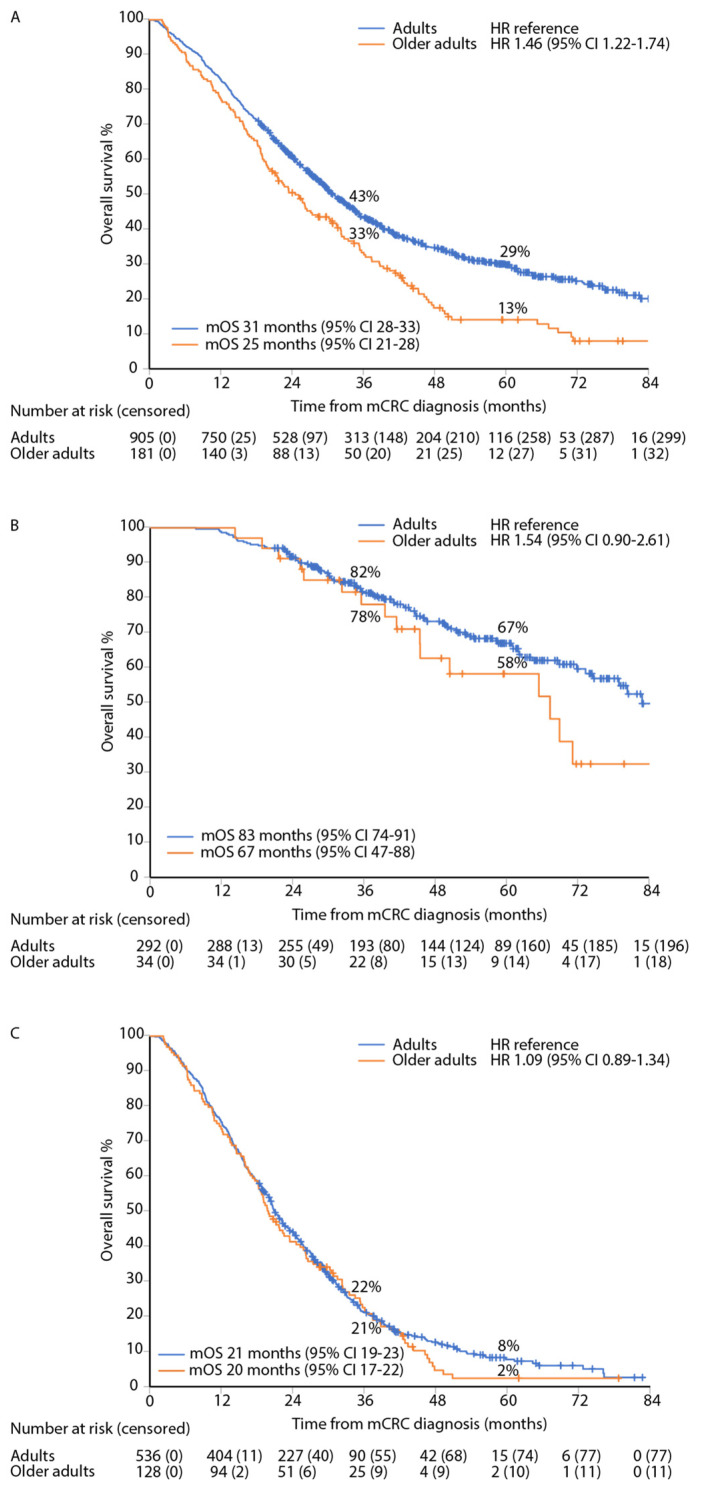
Overall OS of adults and older adults in the whole cohort (**A**), in R0–1 resected (**B**), and in systemic therapy only (**C**).

Per treatment group, there were no statistically significant differences in mOS between older adults and adults. For R0/1-resected patients, mOS was 67 versus 83 months (Figure 4B), for R2/LAT, 32 versus 41 months, and for patients with ‘systemic therapy only’, 20 versus 21 months, respectively (Figure 4C). The mOS for all patients was 25 months (95% CI 21–28) in older adults and 31 months (28–33) in adults, reflecting the different proportions of metastasectomy/LAT between older adults and adults (univariate HR 1.46 (95% CI 1.22–1.74); Figure 4A).

Three-year OS-rates for R0/1-resected were 78% (95% CI 64–93) in older adults and 82% (77–86) in adults (*p* = 0.636), and 5-year OS-rates were 58% (40–77) and 67% (61–73, *p* = 0.383), respectively.

### 3.6. Univariate and Multivariable Regression Analyses of OS

In the older adults, age, ECOG PS, no resection/LAT, right-sided primary, no upfront surgery of primary, and *RAS* or *BRAF* mutation were significantly associated with poorer OS, whereas Charlson comorbidity index was not significant in univariate Cox regression analysis (Table 4). Adults showed similar results, but age was not significant.

In Cox multivariable regression analysis ECOG PS, no resection/LAT, no surgery of primary, and *BRAF* mutation remained statistically significant in both groups (Table 4). For older adults, in addition, age was significant, and for adults, sidedness of primary remained statistically significant (Table 4).

### 3.7. Surgical Complications and Adverse Events

Generally, there were no differences in incidence of postoperative complications, as per resection/LAT, between the age groups (Table S1). Wound complications were slightly more common among older adults (13% vs. 8%, *p* = 0.175). The 30-day and 90-day mortality were both 0.0% among older adults and 0.3% and 0.5%, respectively, among adults.

Regarding systemic therapy-related toxicity, including neoadjuvant, adjuvant, and ‘systemic therapy only’, the only significant difference in grade 3–4 toxicity was less diarrhea among older adults than in adults (Table S2). There were more grade 1–2 anemia and elevated creatinine among older adults compared with adults, whereas less grade 1–2 elevated transaminases, stomatitis, skin toxicity, infections, and neuropathy were noted in the older adults.

### 3.8. HRQoL Index Scores

HRQoL questionnaires were answered by 47 (87% of invited) patients among older adults and 397 (94% of invited) adults. Baseline demographics of patients included and invited but not responding in the HRQoL substudy separately for older adults and adults are presented in Table S3.

Of the older adults participating in the HRQoL study, 20 had had liver resection/LAT, 3 had lung resection/LAT, 1 had cytoreductive surgery, and 1 had resection of subcutaneous metastases. Fourteen older adults (70%) were still in a curative treatment phase when returning questionnaires, of which 13 had had liver resection/LAT (65% of older adults with liver resection/LAT), 3 had lung resection/LAT (100%), 1 had cytoreductive surgery (100%), and/or 1 had resection of subcutaneous metastases (100%). Six of the resection/LAT patients answered the questionnaires only in non-curative treatment phases, that is, after having a non-re-resectable relapse.

**Figure 5:**
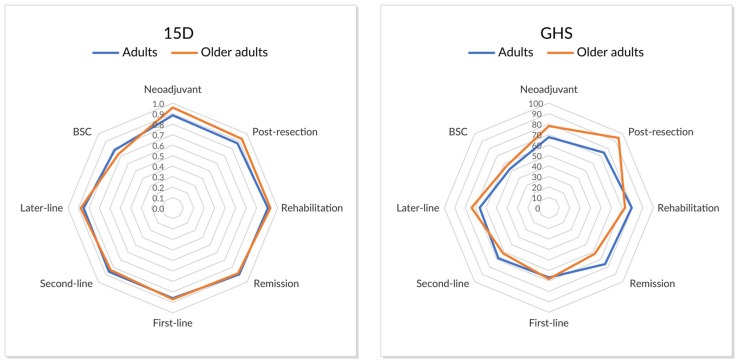
Index scores of 15D and QLQ-C30 global health score (GHS) in adults (n = 52–148 per phase) and older adults (n = 4–21 per phase) in the different treatment phases.

Statistically non-significant minimal clinically important differences (MID) for 15D (≥|0.015|) and QLQ-C30 GHS (≥|5|) were noted during the curative treatment phases of neoadjuvant treatment, postresection (within 6 months from resection), and rehabilitation (6–18 months from resection, only 15D), favoring older adults (caveat n = 4–7 per phase, 14 patients in total) over adults (n = 52–125; Figure 5 and Table 5). During the remission phase (>18 months after resection), no difference was noted for 15D, and a non-significant MID for GHS favoring adults.

During non-curative palliative systemic therapy first-line treatment phases, no significant MID differences were noted (Table 5). When comparing the six resected and later relapsed patients with those never resected during first-line treatment, a non-significant MID was noted for both 15D and GHS (mean 0.884 vs. 0.802, and 83.3 vs. 63.9, respectively). Some non-significant MID differences between older adults and adults were noted during second- and later-line treatment as well as for BSC, though often divergent for 15D and GHS indexes.

For older adults during curative treatment phases, mean 15D indexes (scale 0–1, with 1.0 being best) of 0.882–0.959 were noted, and during palliative systemic treatment—0.836–0.877. For adults, the corresponding results were 0.872–0.907 and 0.849–0.860, respectively (Table 5). For older adults, GHS scores (scale 0–100, with 100 being best) of 62–94 during curative phases and 62–73 during palliative phases were noted, and for adults— 68–79 and 66–68, respectively.

In older adults with curative treatment, 15D indexes decreased during longer follow-up which was not seen as clearly in adults (Table 5). The same was also seen in GHS with some exceptions. In the non-curative groups, there were no clinically significant differences between the first-, second- and later-line treatment in older adults or adults. Lowest values in both age groups were during the BSC phase.

### 3.9. HRQoL Profile, Functioning, and Symptom Scales

There is a trend for better 15D profile scores in older adults compared with adults during neoadjuvant and postresection phases, with no major differences during rehabilitation and remission phases (Figure S1).

No significant differences in physical role; emotional, cognitive, social functioning, or the other functioning profile scales from QLQ-C30 or QLQ-CR29, apart from sexual interest, were noted in older adults compared to adults (Figure S2).

**Figure 6:**
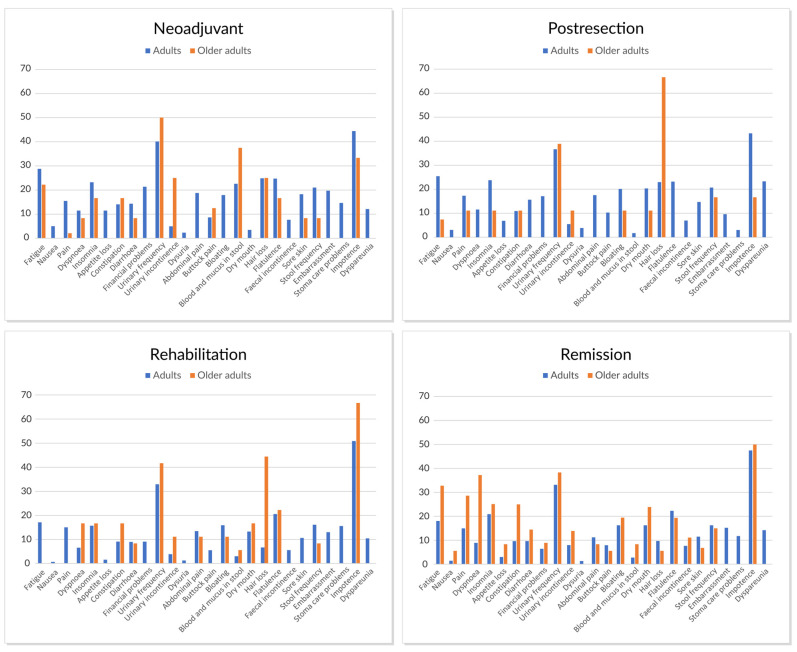
The mean health-related quality of life measured with QLQ-C30 and QLQ-CR29 symptom profile scales before (**A**) and after metastasectomy (**B**), during rehabilitation (**C**), and remission (**D**) in older adults (n = 4–7) and adults (n = 52–125).

For symptom scales based on QLQ-C30 and CR-29, no significant differences apart from hair loss were noted between older adults and adults (Figure 6). The most common symptoms with a mean score over 30 during curative (neoadjuvant/conversion, metastasectomy, adjuvant) treatment phases in older adults were impotence, hair loss, urinary frequency, and dry mouth compared to impotence and urinary frequency in adults.

In long-term follow-up (remission phase >18 months from resection), the most common complaints were impotence, urinary frequency, dyspnea, and fatigue in older adults, and impotence and urinary frequency in adults.

## 4. Discussion

The present study reports that by centralized assessment it is possible to achieve high resection rates with impressive survival in older adults without jeopardizing HRQoL, but local assessment underestimates resectability in this patient group.

Prognosis of mCRC has improved significantly during the past few decades as new chemotherapeutical and biological agents have evolved, but still, the only curative option for metastatic disease is radical surgery of the primary tumor and all metastatic sites [2]. The evidence for liver, lung, or peritoneal resections comes from patient series, mainly multi- or single-center studies, but a few randomized or population-based series have been published (review in [16]). However, less than 25% of patients in clinical studies are over 65 years old, whereas the RAXO prospective study included 55%, and those who were enrolled typically had fewer functional impairments and comorbidities than seen in population-based studies [22,23]. This is true also for the metastasectomy series [5,24,25].

According to our results, even though R0/1-resections were performed less often in older adults than in adults, probably due to impaired functional status and comorbidities, high technical resectability turned into high resection and/or LAT rates also in older adults. Patients were rediscussed in an experienced MDT based on full knowledge of the clinical situation; only technical resectability was assessed centrally, and referred for resection/LAT to a tertiary center according to expertise. Older adults who were assessed to be upfront resectable, significantly more often had LAT than R0/1-resection compared with adults. This emphasizes that older adults with technically resectable metastases are not necessarily surgically fit but could still successfully undergo curative intent LAT, for example, thermoablation or SBRT. The chance of being converted to resectable was also lower for older adults than for adults (10% vs. 19%, respectively). These findings are in line with a Norwegian population-based study where patients under 60 years old had the highest resection rate of 31% for CRC liver metastases with rates declining to 18% for 75–79 year olds and <5% for over 80 year olds [26]. Other population-based analyses reported that liver and lung metastasectomies were performed more than twice as often for <80 year olds than for those >80 years [27], and that patients resected for CRC liver metastases were younger than those receiving systemic therapy only or LAT, who again were younger than those receiving BSC only [28].

Regardless of fewer resections in older adults, we show impressive 3-year OS-rates after R0/1-resection in both age groups (partly attributed to the high re-resection rate with mean 1.6–1.7 procedures per patient); 78% in older adults versus 82% in adults, and this compares well with the literature showing 3-year OS-rates of 34–57% in older adults versus 44%-60% in adults [5,7,25]. As mOS in clinical trials with highly selected adult patients treated with systemic therapy currently reaches approximately 30 months, surgery with curative intent appears to more than double survival also in older adults (mOS 67 months) in the present real-life study. Outcome in older adults is thus slightly inferior to adults, but a clinically meaningful survival advantage is seen with metastasectomy also in older adults.

Compelling evidence supports that more than the actual chronological age of the patient, the quality of aging, comorbidities, and the functional status define treatability [24,29,30]. Many studies conclude that age per se should not be a contradiction for treatment in mCRC [24]. In this study, ECOG, that is, performance status, not the comorbidity index, was a predictor of outcome both in adults and older adults further supporting the notion that performance status may be a better indicator of treatability than number of comorbidities [31,32]. Therefore, older adults with good performance status and co-morbidities without contraindications for active chemotherapy or metastatic surgery should be offered both good management of comorbidities and active cancer therapy.

Present guidelines recommend that all mCRC patients, regardless of age, should be evaluated in MDTs [2,33], but this is not always standard practice in everyday clinics. A French study reported that older adults were less likely to be presented in MDTs [12]. A German study reported that, especially in patients ≥70 years old, MDT assessment had significant impact on the treatment choice, and more patients were resected and received neoadjuvant treatment if assessed [34]. In line with these studies, we here report a high rate of discrepancy in the assessment of technical resectability between centralized and local assessment in adults, and this underestimation is even more pronounced in older adults reflecting the possible undertreatment in this patient group. This was seen most often in patients assessed by an oncologist or a bowel MDT, but also by local organ-specific surgeons and MDTs, which may reflect age discrimination. Resectability is known to be highly dependent on the experience and the skills of the MDT members and the surgeons performing the metastasectomies [16,35], and thus, the differences between local and centralized MDTs can be explained by lack of experience among general CRC surgeons or oncologists compared to organ-specific specialists [36]. Today’s cancer care is complex with many treatment options, and the presence of comorbidities and frailty further increases this complexity. Therefore, we highly recommend that technical resectability of mCRC patients, regardless of age, should be assessed by an experienced MDT including geriatricians at a high-volume academic center. Though the evidence supporting the use of CGA is already convincing in oncology [37,38,39], the lack of geriatricians is the most frequent reason for not using CGA in daily routine [40]. If a geriatrician is not available, at least a geriatric screening should be implemented to identify those in risk of frailty [41,42,43].

We did not note any differences in postoperative mortality (0.0 vs. 0.5%) or morbidity between older adults and adults, contrary to some previous findings. A meta-analysis of 16 studies compared older adults versus adults undergoing liver surgery for mCRC and showed higher postoperative mortality (RR 2.53, varying from 0 to 8% in the studies) and decreased overall survival (RR 1.53) in older patients, whereas no differences in operative outcomes, postoperative complications, or disease-free survival were found compared to adults [24], which is in line with a population-based retrospective cohort of over 75 year olds [6]. Morbidity, mortality, and OS after pulmonary metastasectomy with lymphadenectomy for elderly patients over 70 years old were comparable to younger patients [44]. Older frail patients have a higher risk of postoperative complications [24,45], and these unfit patients would likely benefit from comprehensive geriatric assessment (CGA) before metastasectomy [46]. Prehabilitation before liver resection was shown to improve cardiopulmonary performance status and QoL in a randomized study and may thus enhance outcomes in older mCRC patients with curative intent treatment [47].

Regardless of the intent of the treatment (neoadjuvant, adjuvant, or palliative systemic therapy), we observed no major differences in adverse events between adults and older adults. Mild to moderate elevated creatinine and anemia were more common among older adults than in adults, but older adults had less neuropathy, diarrhea, and stomatitis, which probably reflects treatment intensity. Treatment-related toxicity has been of concern in older adults, and even though there are some studies reporting increased toxicity and more hospitalization in older adults [48], our findings are in line with previously published studies showing no increased toxicity in older adults, especially when modifying treatment intensity [49,50,51,52]. Avoiding severe toxicity and hospitalization is of special importance in older adults, especially if frail, as they are at major risk of permanent decline of the functional capacity in daily living [49,53].

HRQoL after metastasectomy for patients with single-site metastases in mCRC has been reported, but separately for older adults only for primary tumor and lung resections. HRQoL declines after liver metastasectomy and recovers 3–12 months thereafter [54,55,56,57], which is in line with our findings. In these liver resection studies, no data are presented separately for older age groups. In a study with colorectal peritoneal metastases undergoing cytoreductive surgery, the patients had, at two years after treatment, comparable HRQoL to the general population, again without reporting separately for older adults [58]. A study with 126 patients who underwent resection of pulmonary metastases from multiple solid primaries reported HRQoL preoperatively and 3 months postoperatively [59]. Older adults (>70 years, n = 28) had a postoperative tendency on decreased GHS (*p* = 0.09) in line with our findings in older adults. These findings after metastasectomy are in line with findings after primary CRC surgery with intermittently reduced HRQoL in older adults, also frail, as in adults [60,61,62].

The HRQoL data after multisite and multiple CRC metastasectomies are scarce [21,63] with no data published separately on older adults. In a small study with multisite metastasectomies, HRQoL measured with GHS declined transiently after the interventions, with recovery within 1 year post resection [63]. In our present study, after multisite, that is, liver, lung, cytoreductive, and other resections/LATs for mCRC, and multiple procedures (mean 1.6–1.7), HRQoL measured with 15D index decreased transiently in postresection phase but recovered thereafter. On the contrary, GHS did not present the same trend. Both 15D and GHS indexes presented MIDs between older adults and adults during curative treatment phases. In patients relapsing after surgery, HRQoL remained good also during non-curative first-line treatment. At remission, when comparing the age groups, the indexes were at the same level. Age alone in primary CRC patients is not associated with HRQoL whether older or younger [14], and even the age group of 90–99 years old community dwellers demonstrated fairly high levels of HRQoL [64]. These results support active evaluation of resectability of multiple and multisite metastases also in older adults, not forgetting the LAT alternatives if older adults are not fit enough for resection.

15D profile scales and QLQ functioning scales showed slightly higher scores for older adults than adults in neoadjuvant and postresection phases, but no major differences in follow-up phases apart from sexual interest, which was lower in older adults than in adults. A prospective study of adults over 70 years old undergoing primary surgery of CRC showed that functionally independent patients experienced a higher HRQoL measured with QLQ-C30 and CR38 than dependent patients, whereas dependent patients reported clinically relevant improvement in the majority of the HRQoL domains, compared to pre-surgery responses [60]. Functioning scales were good in long-term survivors after liver resection, without separation for age groups [56].

Symptom burden measured by QLQ-C30 and QLQ-CR29 presented statistically significant differences only for hair loss postoperatively in older adults compared with adults. During curative (neoadjuvant/conversion, metastasectomy, adjuvant) treatment phases and thereafter, both age groups complained of impotence and urinary frequency, and older adults complained of dyspnea and fatigue as well. This is in line with findings for patients after pulmonary resection [59]. In the long term, the complaints may be more dependent on aging and surgery of the primary than on the treatment of metastases.

We demonstrate a good overall survival of 20–21 months both in adults and older adults treated with palliative systemic therapy only. Our results are in line with randomized trials demonstrating that older adults derive equal benefit from systemic treatment as adults, but older adults are underrepresented in these trials [49,65,66]. Prospective real-life data are scarce [67], and to the best of our knowledge, our study is one of the few ones reporting OS in unselected older adults treated with systemic chemotherapy in a real-life and population-based setting. We observed no significant differences in HRQoL between adults and older adults which is line with a retrospective analysis from the randomized CAIRO trials [49]. Results from the prospective NORDIC9 trial showed similar HRQoL in dose-reduced combination therapy as in single-agent chemotherapy [51]. Our results underlie that by offering systemic therapy to older adults with non-resectable metastatic disease, good survival can be achieved also in this age group, and HRQoL can be maintained.

The major limitation of the present study is that very few older adults in the curative intent group answered HRQoL questionnaires (14 during curative phases and 6 thereafter), and, thus, all our HRQoL results should be interpreted with great caution. The questionnaires were also recorded cross-sectionally and not longitudinally at prespecified timepoints. The HRQoL substudy started in 2017 when the RAXO-study per se had been ongoing since 2012. This led to a risk of guarantee-time bias. It is noteworthy that only treatable patients were included and BSC patients were excluded. Thus, our study population does not represent the older adults as a whole. Treatability was assessed clinically with ECOG PS, organ function assessment, comorbidities, and willingness to have systemic treatment and metastasectomy. CGA, defining functional status, is now, by many, considered as standard of care assessment for older adults with frailty, but it was not routine in 2012–2018, when this study was conducted [37,38,39,46]. The strengths of this study include the prospective setting with a relatively large national real-life population, description of the background population, multiple and multisite metastases, the centralized assessment by an experienced MDT enabling high resection/LAT rates also in older adults over 75 years old, and mature data with no patients lost to follow-up.

From the doctor’s perspective, data for OS of older adults are generally inferior to that of adults [5,9,24], especially population-based as in the RAXO data collection study, possibly introducing nihilism when offering treatment to older adults without significant comorbidities. However, this is partly explained by the smaller proportion of patients having metastasectomies [26] (23 vs. 8%), that we often forget the toolbox of LATs which offers an alternative when metastasectomy is not feasible [2], and that fewer patients receive active chemotherapy (71% vs. 33%). OS as opposed to cancer-specific survival is also affected by non-CRC deaths, and more abundant in older adults [25]. Older adults also have tumor characteristics with inferior outcome, such as more right-sided primaries, non-favorable metastatic sites, *BRAF* mutations, deficient mismatch repair, etc. [68,69,70]. Tailoring of systemic therapy and inclusion of CGA in the treatment path is also considered cumbersome [71].

From the older adult patient’s perspective, shared decision making is important. A doctor’s anticipation of poorer outcome and more toxicities in older adults may lead to underestimation of the value of proper MDT assessment and treatment options in the discussions with older patients. Given the evidence, treatable older adults have similar outcomes as adults when actively treated, without compromising HRQoL [59,61,72], and also after multisite and multiple metastasectomies according to this study. Of notice is also a better HRQoL in older adults during curative treatment phases than during systemic treatment. The duration of systemic treatment is also significantly shorter in curative-intent treatment—usually 6 months of perioperative treatment. Based on the increasing evidence for reasonable morbidity, mortality, and adverse events from therapy in treatable older adults, we should not exclude resectable patients from metastasectomy and/or LAT, which have clearly better outcomes than systemic therapy.

## 5. Conclusions

In conclusion, complete curative resection of mCRC leads to excellent survival even in older adults with a high average HRQoL in a small number of patients participating in the HRQoL substudy. Fit patients with mCRC should, regardless of chronological age, be actively evaluated by a centralized MDT and offered curative intent surgical treatment.

## Figures and Tables

**Table 1:** Baseline characteristics.

		Adults		Older Adults		*p*-Value
		n = 905 (83%)		n = 181 (17%)		
Age	Median (range)	64	(24–75)	78	(75–90)	**<0.001**
Sex	Male	549	61%	107	59%	0.698
	Female	356	39%	74	41%	
ECOG	PS 0	266	29%	29	16%	**<0.001**
	PS 1	500	55%	100	55%	
	PS 2–3	139	15%	52	29%	
Charlson comorbidity index *	0	719	79%	115	64%	**<0.001**
	1 to 2	179	20%	65	36%	
	3 to 5	7	1%	1	1%	
Second cancer	Non-colorectal cancer	106	12%	37	20%	**0.002**
Presentation of metastases	Synchronous	632	70%	104	57%	**0.001**
	Metachronous	273	30%	77	43%	
Primary location	Right colon	247	27%	63	35%	0.127
	Left colon	338	37%	58	32%	
	Rectum	316	35%	58	32%	
	Multiple	4	0%	2	1%	
Surgery of primary	Operated upfront	592	65%	133	74%	**0.035**
	Not operated or later	313	35%	48	27%	
Number of metastatic sites	1 site	497	55%	89	49%	0.153
	2 sites	255	28%	64	35%	
	3 to 6 sites	153	17%	28	16%	
Liver metastases	Liver-limited	365	40%	65	36%	0.345
	Liver and extrahepatic	310	34%	72	40%	
Lung metastases	Lung-limited	56	6%	10	6%	**0.048**
	Lung and extrapulmonary	207	23%	57	32%	
Other metastases	Peritoneal metastases	144	16%	31	17%	0.685
	Distant lymph nodes	235	26%	40	22%	0.275
	Other metastases	153	17%	30	17%	0.913
Estimated glomerular	≥90 mL/min/1.73 m^2^	463	52%	17	10%	**<0.001**
filtration rate	60–89 mL/min/1.73 m^2^	343	38%	79	44%	
	30–59 mL/min/1.73 m^2^	84	9%	82	46%	
	<30 mL/min/1.73 m^2^	4	0%	1	1%	
Mutational status	*RAS* +/− *BRAF* wt	365	40%	63	35%	0.169
	*RAS* mt	441	49%	98	54%	
	*BRAF* mt	82	9%	11	6%	
	Not tested	17	2%	9	5%	-
Anemia	Hemoglobin <11 g/dL	163	18%	25	14%	0.173
Leukocytosis	Leukocytes >10^9^/L	163	18%	22	12%	0.056
Thrombocytosis	Thrombocytes >400^9^/L	257	28%	35	19%	**0.012**
Hypoalbuminemia	Albumin <30 g/L	90	16%	14	16%	0.915
Alkaline phosphatase	>105 U/L	318	35%	55	31%	0.211
Carcinoembryonic antigen	>5 µ/L	622	70%	136	76%	0.110
Cancer antigen 19-9	>26 kU/L	301	55%	44	51%	0.481

* Charlson comorbidity index excludes 6 points for metastatic cancer as it would be the same for all patients. Statistically significant *p*-values in bold.

**Table 2:** Resections and/or LAT in adults and older adults.

	Adults		Older Adults		*p*-Value
	905	%	181	%	
Liver resections/LAT	279	31%	37	20%	0.005
Lung resection/LAT	74	8%	7	4%	0.044
Cytoreductive surgery	44	5%	4	2%	0.113
Local, lymphadenectomy, gynecologic, urologic, or other	66	7%	9	5%	0.261

**Table 3:** Treatment intent in resected/LAT patients and Systemic therapy only/best supportive care (BSC) patients.

	Metastasectomy and/or LAT					Systemic Therapy Only or BSC Only				
	Adults		Older Adults		*p*-Value	Adults		Older Adults		*p*-Value
	354	39%	45	25%		551	61%	136	75%	
Metastasectomy only	36	10%	6	13%	0.515					
Adjuvant	236	67%	27	60%	0.374					
Neoadjuvant	136	38%	21	47%	0.286	23	4%	11	8%	0.059
Conversion	126	36%	14	31%	0.553	86	16%	20	15%	0.794
Disease control						427	77%	97	71%	0.130
Best supportive care						15	3%	8	6%	0.067

**Table 4:** Cox univariate (**A**) and multivariable (**B**) models of prognostic factors for OS in adults and in older adults.

A		Adults (n = 886)					Older Adults (n = 171)				
Univariate		HR	95% CI			*p*-Value	HR	95% CI			*p*-Value
Age (years)		1.00	1.00	-	1.01	0.313	1.12	1.06	-	1.17	<0.001
ECOG	PS 0	1.00				<0.001	1.00				0.001
	PS 1	1.72	1.41	-	2.10	<0.001	1.79	1.09	-	2.93	0.021
	PS 2–3	3.48	2.70	-	4.49	<0.001	2.69	1.57	-	4.63	<0.001
Treatment groups	R0–1 resection	1.00				<0.001	1.00				<0.001
	R2 resection/LAT	2.53	1.72	-	3.73	<0.001	2.63	1.12	-	6.18	0.026
	Systemic therapy only	6.75	5.35	-	8.52	<0.001	5.62	3.24	-	9.75	<0.001
Charlson comorbidity	0	1.00				0.241	1.00				0.583
index	1–2	1.16	0.95	-	1.42	0.135	1.19	0.84	-	1.68	0.325
	3–5	1.47	0.61	-	3.55	0.393	0.76	0.11	-	5.50	0.789
Primary tumor	Left-sided	1.00				<0.001	1.00				0.008
location	Right-sided	1.67	1.40	-	1.99	<0.001	1.60	1.13	-	2.26	0.008
Primary tumor	Yes	1.00				<0.001	1.00				0.041
operated	No	2.04	1.73	-	2.41	<0.001	1.49	1.02	-	2.18	0.041
Mutational status	RAS +/− BRAF wt	1.00				<0.001	1.00				<0.001
	RAS mt	1.24	1.04	-	1.48	0.014	1.05	0.73	-	1.52	0.793
	BRAF mt	2.66	2.00	-	3.52	<0.001	2.15	1.05	-	4.42	0.037
	Not tested	0.28	0.10	-	0.75	0.012	6.73	2.92	-	15.48	<0.001
B											
Multivariable											
Age (years)		0.99	0.99	-	1.00	0.238	1.07	1.01	-	1.13	0.016
ECOG	PS 0	1				<0.001	1				0.016
	PS 1	1.43	1.17	-	1.75	0.001	1.58	0.96	-	2.63	0.075
	PS 2–3	2.46	1.88	-	3.21	<0.001	2.28	1.29	-	4.02	0.004
Treatment groups	R0–1 resection	1				<0.001	1				<0.001
	R2 resection/LAT	2.23	1.51	-	3.32	<0.001	2.76	1.17	-	6.51	0.020
	Systemic therapy only	5.58	4.38	-	7.10	<0.001	4.66	2.65	-	8.18	<0.001
Charlson comorbidity	0	1				0.856	1				0.587
index	1–2	1.04	0.85	-	1.27	0.709	0.82	0.56	-	1.21	0.327
	3–5	1.22	0.50	-	2.99	0.663	0.67	0.09	-	4.95	0.691
Primary tumor	Left-sided	1				<0.001	1				0.352
location	Right-sided	1.70	1.39	-	2.07	<0.001	1.21	0.81	-	1.83	0.352
Primary tumor	Yes	1				<0.001	1				0.019
operated	No	1.57	1.32	-	1.87	<0.001	1.65	1.09	-	2.51	0.019
Mutational status	RAS +/− BRAF wt	1				0.026	1				0.001
	RAS mt	1.14	0.96	-	1.37	0.139	0.96	0.65	-	1.44	0.855
	BRAF mt	1.45	1.06	-	1.97	0.020	2.70	1.24	-	5.88	0.012
	Not tested	0.45	0.16	-	1.21	0.113	3.71	1.53	-	9.01	0.004

**Table 5:** Mean HRQoL scores and difference = Δ between means of the two age groups (Positive delta indicates better QoL in older adults). Minimal clinically important difference (MID) and statistical differences are bolded: 15D: ≥|0.015|, Quality of Life Questionnaire (QLQ)-C30 Global Health Score (GHS) ≥ |5|.

	Adults				Older Adults					
	n = 397	Mean	SD	95% CI	n = 47	Mean	SD	95% CI	∆	*p*-Value
**15D**										
Neoadjuvant	52	0.884	0.097	0.857–0.911	4	0.959	0.066	0.853–1.064	**0.075**	0.056
Post resection	54	0.872	0.091	0.846–0.896	4	0.932	0.055	0.845–1.019	**0.060**	0.209
Rehabilitation	55	0.907	0.071	0.887–0.925	5	0.931	0.055	0.863–0.999	**0.023**	0.514
Remission	125	0.896	0.091	0.879–0.912	7	0.882	0.111	0.779–0.985	−0.014	0.831
First-line	148	0.860	0.090	0.850–0.880	21	0.870	0.080	0.830–0.900	0.010	0.937
Second-line	104	0.859	0.090	0.842–0.877	10	0.836	0.071	0.785–0.877	**−0.023**	0.356
Later-line	93	0.849	0.097	0.829–0.869	12	0.877	0.072	0.831–0.923	**0.027**	0.435
Best supportive care	22	0.782	0.116	0.730–0.833	13	0.730	0.132	0.650–0.810	**−0.052**	0.302
**GHS**										
Neoadjuvant	52	67.5	20.7	61.3–73.7	4	78.1	11.5	59.9–96.4	**10.66**	0.366
Post resection	54	74.5	17.7	69.1–79.0	4	94.4	9.6	70.5–118.3	**19.90**	**0.037**
Rehabilitation	55	79.2	15.0	74.9–83.1	5	72.9	17.2	45.6–100.3	**−6.30**	0.524
Remission	125	76.1	18.6	72.8–79.5	7	62.2	20.3	40.9–83.5	**−13.91**	0.055
First-line	148	66.6	18.2	63.1–70.1	21	68.1	11.9	62.6–73.7	1.54	0.712
Second-line	104	68.3	18.2	64.5–72.0	10	61.7	16.8	49.7–73.7	**−6.60**	0.234
Later-line	93	66.1	17.8	62.4–69.8	12	73.8	16.8	63.7–84.0	**7.73**	0.129
Best supportive care	22	52.6	19.6	43.7–61.5	13	56.8	23.1	42.9–70.8	4.26	0.576

Four curative treatment phases were identified: neoadjuvant (including more intense conversion therapy) before an R0/1-resection/LAT, post resection/LAT (including adjuvant therapy) during the first 6 months after the R0/1-resection/LAT, rehabilitation (without treatment 6–18 months from an R0/1-resection/LAT or complete response to systemic therapy only), and remission (disease-free for more than 18 months from the last metastasectomy/LAT or complete response). The non-curative treatment phases were: first-line, second-line, and later-line (maximum 8 lines) systemic treatment—whether the intention was to allow subsequent metastasectomy/LAT or not, but it was not reached—and best supportive care (after active oncological treatments were permanently stopped).

## Data Availability

The data collected for this study can be made available to others in a de-identified form after all primary and secondary endpoints have been published, in the presence of a data transfer agreement, and if the purpose of use complies with Finnish legislation. Requests for data sharing can be made to the corresponding author, including a proposal that must be approved by the steering committee.

## Supplementary Materials

The following supporting information can be downloaded at: https://www.mdpi.com/article/10.3390/jcm12103541/s1, Table S1: Postoperative morbidity within 8 weeks from each resection and/or local ablative therapy (LAT) and mortality at 30 or 90 days for procedure for adults (588 procedures in 354 patients) and older adults (72 procedures in 45 patients); Table S2: Adverse events during systemic therapy as neoadjuvant/conversion, adjuvant, and palliative. *p*-values indicate statistical significance per row; Table S3: Patient demographics for patients included in the HRQoL substudy and patients invited but not responding (did not return questionnaires); Figure S1: 15D Profile scale results during curative treatment phases in adults (n = 52–125) and older adults (n = 4–7); Figure S2: The mean health-related quality of life with QLQ-C30 and QLQ-CR29 functioning profile scales in adults (n = 52–125) and older adults (n = 4–7) during curative treatment phases.
