# Supplementary material for: Resectability, Resections, Survival Outcomes, and Quality of Life in Older Adult Patients with Metastatic Colorectal Cancer (the RAXO-Study)
Source: J Clin Med. 2023 May 18;12(10):3541. doi: 10.3390/jcm12103541 (PMC10218996; doi:10.3390/jcm12103541)
Supplement: Supplementary file 1 [file jcm-12-03541-s001.zip › jcm-2235038-supplementary.pdf]

Table S1. Postoperative morbidity within 8 weeks from each resection and/or local ablative therapy (LAT) and mortality at 30 or 90-days for procedure for adults (588 procedures in 354 patients) and older adults (72 procedures in 45 patients)

		Adults 588 procedures		Older adults 72 procedures		P-value
Any complication	No	397	68%	47	65%	0.702
	Yes	191	32%	25	35%	
Postoperative bleeding	No	578	98%	71	99%	0.845
	Yes	10	2%	1	1%	
Any wound complication	No	542	92%	63	88%	0.175
	Yes	46	8%	9	13%	
Postoperative infection	No	470	80%	53	74%	0.297
	Confirmed	89	15%	16	22%	
	Suspected	29	5%	3	4%	
Other complication	No	490	83%	62	86%	0.805
	Yes	98	17%	10	14%	
30-day mortality after metastasectomy	No	586	100%	72	100%	0.620
	Yes	2	0.3%	0	0.0%	
90-day mortality after metastasectomy	No	585	99%	75	100%	0.544
	Yes	3	0.5%	0	0.0%	

Table S2. Adverse events during systemic therapy as neoadjuvant/conversion, adjuvant, and palliative. P-values indicates statistical significance per row

		Adults 867 (83%)		Older adults 173 (17%)		P-Value
Anaemia	Gr 1-2	685	76 %	148	84 %	0.035
	Gr 3-4	30	3 %	4	2 %	
Leucocytopenia	Gr 1-2	277	31 %	46	26 %	
	Gr 3-4	61	7 %	11	6 %	
Neutropenia	Gr 1-2	256	29 %	42	24 %	
	Gr 3-4	245	27 %	41	24 %	
Thrombocytopenia	Gr 1-2	373	42 %	64	37 %	
	Gr 3-4	19	2 %	5	3 %	
Proteinuria	Gr 1-2	30	3 %	6	3 %	
	Gr 3-4	1	0 %	1	0 %	
Transaminases elevated	Gr 1-2	397	45 %	42	24 %	<0.001
	Gr 3-4	56	6 %	9	5 %	
Creatinine elevated	Gr 1-2	186	21 %	64	37 %	<0.001
	Gr 3-4	6	1 %	1	0 %	
Diarrhoea	Gr 1-2	345	38 %	63	35 %	
	Gr 3-4	78	9 %	6	3 %	0.015
Constipation	Gr 1-2	259	29 %	45	25 %	
	Gr 3-4	14	2 %	1	0 %	
Stomatitis	Gr 1-2	191	21 %	24	13 %	0.016
	Gr 3-4	9	1 %	2	1 %	
Mucositis	Gr 1-2	198	22 %	31	17 %	
	Gr 3-4	9	1 %	1	1 %	
PPE or Skin	Gr 1-2	376	42 %	59	33 %	0.025
	Gr 3-4	43	5 %	5	3 %	
Infections	Gr 1-2	106	12 %	11	6 %	0.026
	Gr 3-4	250	28 %	40	22 %	
Fatigue	Gr 1-2	172	19 %	39	22 %	
	Gr 3-4	7	1 %	2	1 %	
Neuropathy	Gr 1-2	526	58 %	58	32 %	<0.001
	Gr 3-4	20	2 %	1	1 %	
Allergic reaction	Gr 1-2	11	1 %	1	0 %	
	Gr 3-4	15	2 %	3	2 %	
Cardiac disorders	Gr 1-2	11	1 %	4	2 %	
	Gr 3-4	20	2 %	2	1 %	
Hypertension	Gr 1-2	49	5 %	5	3 %	
	Gr 3-4	17	2 %	1	1 %	
Thromboembolic event	Gr 1-2	4	0 %	1	0 %	
	Gr 3-4	64	7 %	16	9 %	
Bowel perforation	Gr 1-2	1	0 %	1	0 %	
	Gr 3-4	8	1 %	1	0 %	
Fistula	Gr 1-2	1	0 %	1	0 %	
	Gr 3-4	10	1 %	1	1 %	
Bleeding	Gr 1-2	20	2 %	2	1 %	
	Gr 3-4	12	1 %	1	0 %	

Table S3. Patient demographics for patients included in the HRQoL substudy and patients invited but not responding (did not return questionnaires)

		Adults ≤75 years					Older adults >75 years				
		Invited, not responding		Included		P-value	Invited, not responding		Included		P-value
		27	6 %	397	94 %		7	13 %	47	87 %	
Sex	Male	20	74 %	231	58 %	0.104	4	57 %	27	57 %	0.988
	Female	7	26 %	166	42 %		3	43 %	20	43 %	
ECOG	PS 0	3	11 %	160	40 %	**<0.001**	0	0 %	12	26 %	0.077
	PS 1	17	63 %	206	52 %		4	57 %	29	62 %	
	PS 2-3	7	26 %	31	8 %		3	43 %	6	13 %	
Comorbidities	No	6	22 %	121	31 %	0.588	1	14 %	6	13 %	0.980
	1 to 3	17	63 %	234	59 %		5	71 %	33	70 %	
	4 to 12	4	15 %	42	11 %		1	14 %	8	17 %	
Second cancer	No	24	89 %	351	88 %	0.940	6	86 %	40	85 %	0.966
	Non-colorectal cancer	3	11 %	46	12 %		1	14 %	7	15 %	
Presentation of metastases	Synchronous	21	78 %	273	69 %	0.326	3	43 %	25	53 %	0.601
	Metachronous	6	22 %	124	31 %		4	57 %	22	47 %	
Primary location	Right colon	11	41 %	93	23 %	**<0.001**	1	14 %	16	34 %	0.152
	Left colon	5	19 %	164	41 %		1	14 %	17	36 %	
	Rectum	10	37 %	140	35 %		5	71 %	13	28 %	
	Multiple	1	4 %	0	0 %		0	0 %	1	2 %	
Surgery primary tumour	Operated upfront	10	37 %	277	70 %	**<0.001**	5	71 %	37	79 %	0.665
	Not operated upfront	17	63 %	120	30 %		2	29 %	10	21 %	
Metastatic sites	1 site	13	48 %	256	65 %	0.064	3	43 %	28	60 %	0.479
	2 sites	7	26 %	96	24 %		3	43 %	17	36 %	
	3 to 6 sites	7	26 %	45	11 %		1	14 %	2	4 %	
Liver metastases	Liver-limited	10	37 %	187	47 %	0.118	2	29 %	23	49 %	0.542
	Liver & extrahepatic	12	44 %	104	26 %		3	43 %	12	26 %	
Lung metastases	Lung-limited	2	7 %	37	9 %	0.481	1	14 %	3	6 %	0.656
	Lung & extrapulmonary	7	26 %	67	17 %		1	14 %	12	26 %	
Other metastases	Peritoneal metastases	5	19 %	54	14 %	0.475	1	14 %	4	9 %	0.623
	Distant lymph nodes	9	33 %	82	21 %	0.120	0	0 %	13	28 %	0.110
	Other metastases	3	11 %	50	13 %	0.822	3	43 %	3	6 %	**0.004**
Estimated glomerular filtration rate	≥90 ml/min/1.73m2	10	37 %	203	52 %	0.263	0	0 %	5	11 %	**0.013**
	60-89 ml/min/1.73m2	15	56 %	156	40 %		6	86 %	26	55 %	
	30-59 ml/min/1.73m2	2	7 %	34	9 %		0	0 %	16	34 %	
	<30 ml/min/1.73m2	0	0 %	0	0 %		1	14 %	0	0 %	
Mutational status	RAS +/- BRAF wt	9	33 %	160	40 %	**0.037**	2	29 %	20	43 %	0.256
	RAS mt	12	44 %	198	50 %		4	57 %	26	55 %	
	BRAF mt	6	22 %	28	7 %		1	14 %	1	2 %	
	Not tested	0	0 %	11	3 %		0	0 %	0	0 %	
Haemoglobin	<11 g/dL	5	19 %	55	14 %	0.501	1	14 %	8	17 %	0.856
Leucocytes	>10 ^9/L	8	30 %	53	13 %	**0.020**	2	29 %	3	6 %	0.059
Thrombocytes	>400 ^9/L	8	30 %	99	25 %	0.587	2	29 %	8	17 %	0.436
Albumin	<30 g/L	6	38 %	29	12 %	**0.004**	1	33 %	2	11 %	0.309
Alkaline phosphatase	>105 U/L	12	46 %	109	28 %	0.044	3	43 %	10	21 %	0.213
Carcinoembryonic antigen	>5 µ/L	22	85 %	244	62 %	**0.022**	6	86 %	38	81 %	0.757
Cancer antigen 19-9	>26 kU/L	10	63 %	116	46 %	0.191	3	75 %	15	68 %	0.786

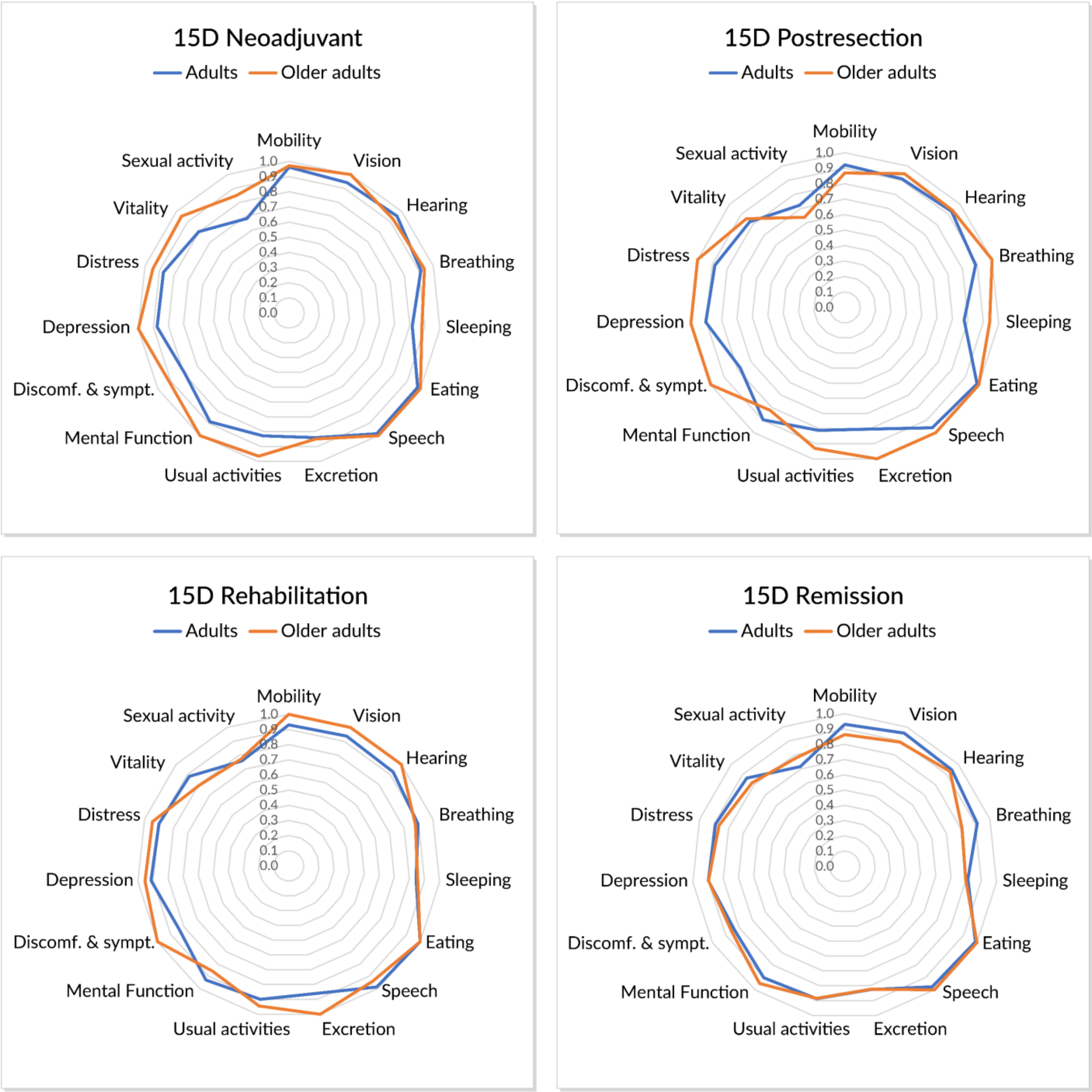

Figure S1. 15D Profile scale results during curative treatment phases in adults (n=52-125) and older adults (n=4-7)

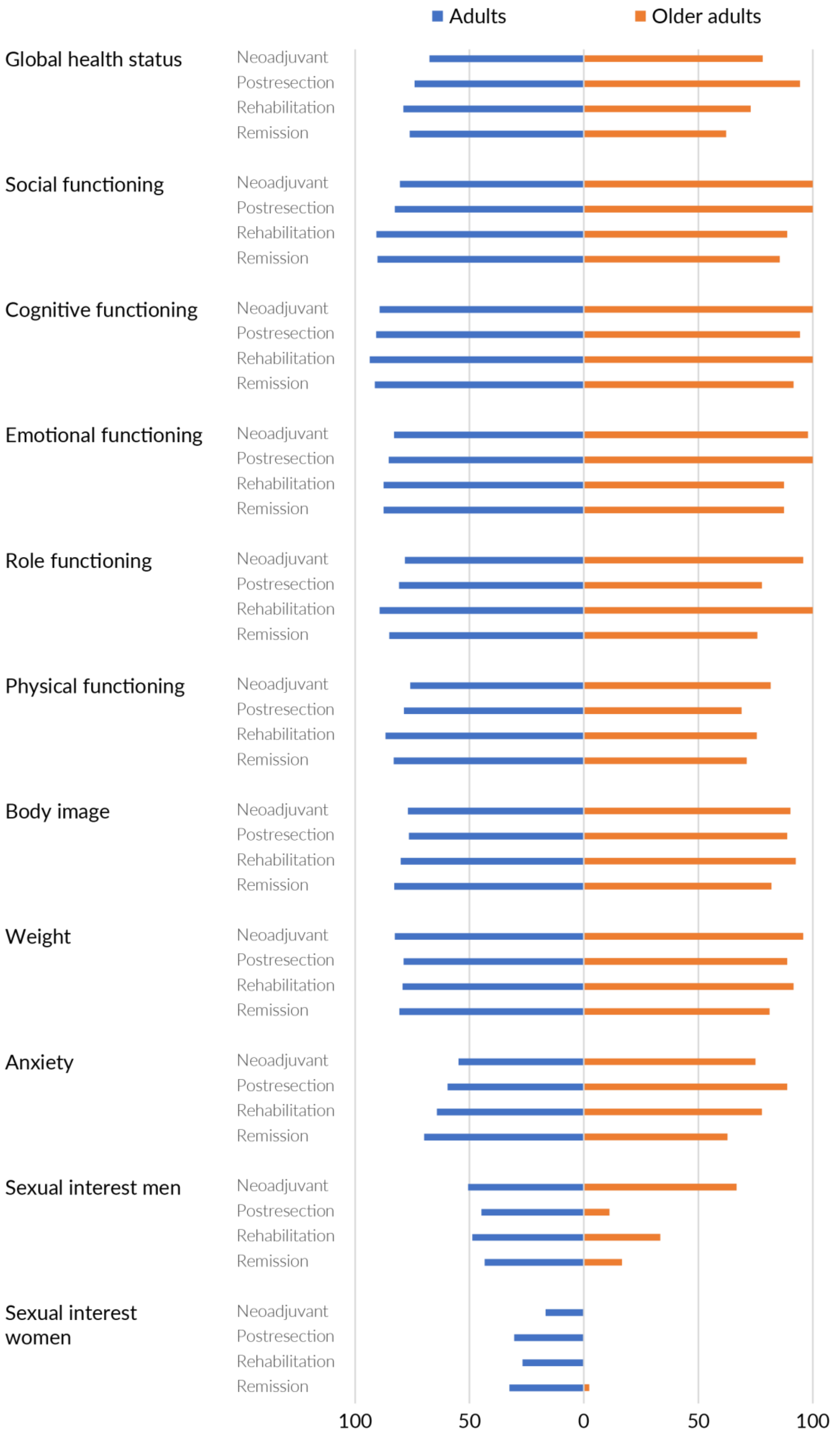

Figure S2.The mean health-related quality of life with QLQ-C30 and QLQ-CR29 functioning profile scales in adults (n=52-125) and older adults (n=4-7) during curative treatment phases
